# A Novel Method to Indirectly Measure Electro-osmotic Drag and Back Diffusion From Total Water Flow Experiments in PEM Fuel Cells

**DOI:** 10.1021/acsomega.5c06100

**Published:** 2026-01-15

**Authors:** Nicholas A. Ingarra, Krzysztof Chris J. Kobus

**Affiliations:** Department of Mechanical Engineering, 6918 Oakland University, Rochester, Michigan 48309, United States

## Abstract

The objective of the research is to quantify the electro-osmotic drag and back diffusion portions of the experimentally measured total water flow across a proton exchange membrane. Part of the deficiency of prior research calculated individual coefficients by forcing trendlines through the origin that implicitly assumes other drivers to be negligible. If one or more other drivers are present, their impact will be lumped in with the intended coefficient measurement. Also, using linear trendlines assumes that the indirectly measured coefficient does not change with current density, which is likely the case for liquids but not for vapor. For fuel cell membranes like Nafion, the electro-osmotic drag and back diffusion coefficients are dependent on the hydration state of the membrane. To overcome this dependency, a higher-order polynomial data fit is used for the total water flow. A methodology of combining the theoretical model with experimental data sets is proposed here to determine the component coefficients at each data point. Applying this method to prior data reveals that other fluid drivers assumed to be negligible were likely not so. A more accurate understanding of electro-osmotic drag and back diffusion in turn enables more accurate prediction of total water flow from each driver, leading to improving water management and reducing the risks of cathode catalyst layer flooding and membrane dry-out.

## Graphical abstract

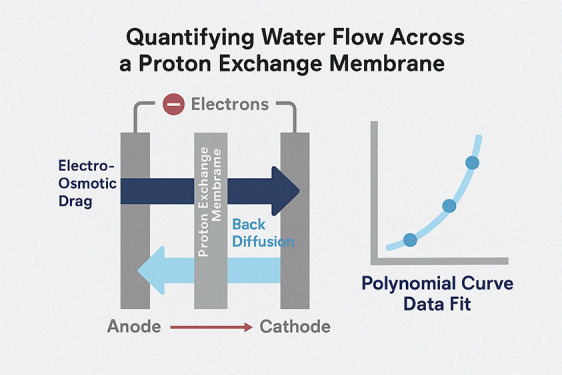

## Introduction

The PEM fuel cell requires precise water management, and it requires the membrane to be hydrated to support electro-osmotic drag, but the membrane cannot be overhydrated because it can cause liquid water to form in the cathode catalysts layer that can lead to flooding and loss of performance. The total water flow across the membrane must be controlled to prevent both the membrane from drying out (which would increase ohmic losses) and flooding (which would increase heat generation and reduce the discharge voltage). According to the Nernst–Planck equation, there are two modes of fluid transport across the membrane: electro-osmotic drag and back diffusion.

The anode (hydrogen) side of the fuel cell requires humidification to support electro-osmotic drag, and this is done by transporting water from the cathode to the anode via Fickian diffusion. The amount of water transported via electro-osmotic drag must be replenished. To aid the back diffusion, a humidifier is placed on the cathode side to humidify the incoming air into the fuel cell. If the electro-osmotic drag coefficient is higher than predicted, then more water will be transported across the membrane, and this will lead to over-humidification of the cathode, which can lead to flooding. If on the other hand the electro-osmotic drag coefficient is less than predicted, insufficient water will be transported across the membrane, and if the cathode humidifier is sized properly, the membrane may dry out. These are main reasons why accuracy in quantifying membrane water transport drivers is required for the best fuel cell operation.

Because of the known uncertainties in fluid driver magnitudes, humidifiers and other components in the balance of the system are designed larger than what would be needed if accuracy was achieved. With better modeling, components such as humidifiers could be reduced or outright eliminated, leading to simpler and more optimized performance.

In electro-osmotic drag or back diffusion experiments where the total fluid flow across the membrane is indirectly measured, this one measurement is a result of all of the fluid drivers present in the experiment. In the case of a fuel cell, there are multiple drivers of water transport, some of which are well-known and some less so. Much of the available experimental data was focused on specific transport coefficients such as electro-osmotic drag (EOD), back diffusion (BD), and, to a lesser extent, thermal osmosis (TO). To quantify the magnitude of the various drivers when more than one is present, conventional researchers have chosen an available empirical model for at least one of these, with the remainder being subscribed to the other driver or drivers; thus, in the case when only EOD and BD are (assumed to be) present,

$$J_{\mathrm{Total}}-J_{\mathrm{EOD}}=J_{\mathrm{BD}}$$ 1

Referring to Figures 1 and 2, the problem here is that there is a large amount of scatter in the available empirical models, as they have been developed by researchers under many different conditions. The different models show different trends, and it is not clear in many instances which model yields the best results under specific operating conditions. With the scatter in the electro-osmotic drag and back diffusion coefficients, in addition to various trends with respect to membrane hydration state, it adds an element of uncertainty to both, as the back diffusion coefficient calculation will be coupled to the electro-osmotic drag coefficient model chosen, or vice versa, as per eq 1. A method is thus called for to calculate the individual contributions without requiring prior empirical models. The current research will show how to determine these component portions of the total water flow without assuming one of these models.

**Figure 1:**
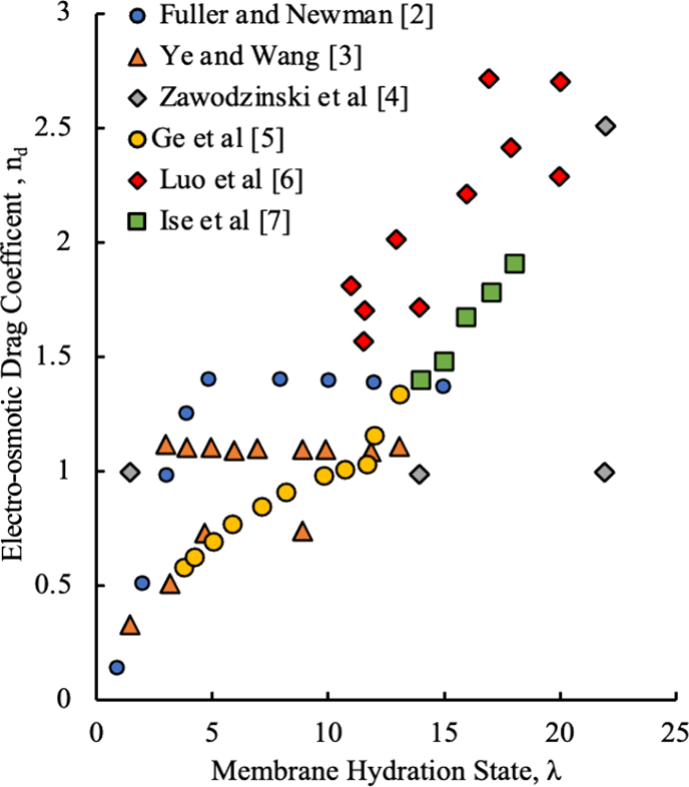
EOD coefficient data from various research efforts.^1–7^

**Figure 2:**
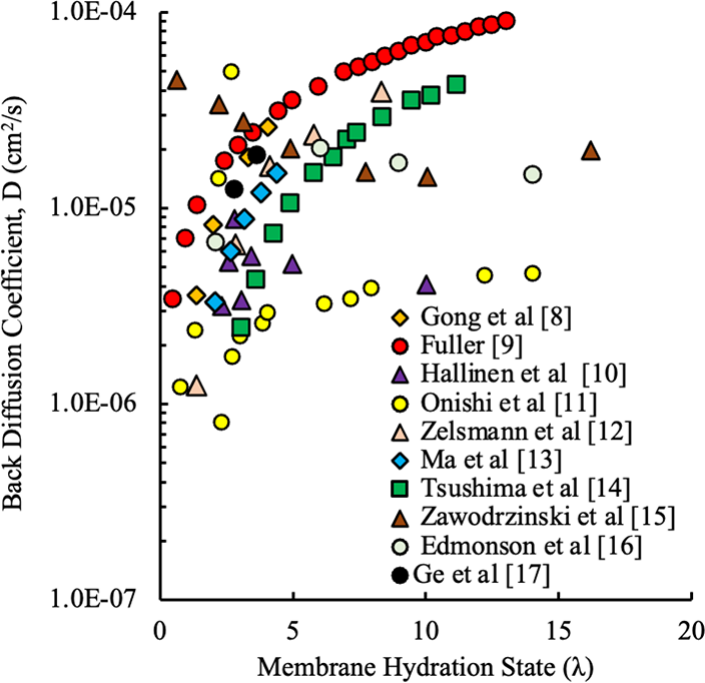
BD coefficient data from various research efforts.^8–17^

In the available literature, the mass flow entering and exiting either side (anode or cathode) of the fuel cell is measured to compute the total water flow crossing the membrane. A typical setup is shown in Figure 3:^18^

**Figure 3:**
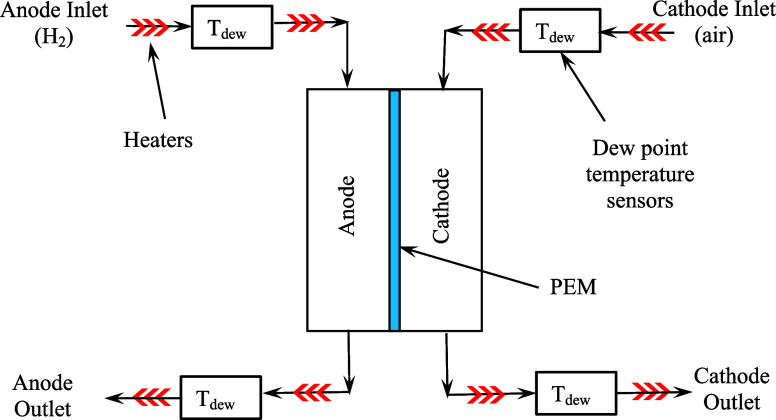
Sample of how total water flow is measured.^18^

The membrane of the fuel cell is a porous medium that allows water to move from the anode to cathode or vice versa. The total water flow can be measured indirectly and calculated through a difference in mass flow into and out of either side of the fuel cell; thus,

$$J_{\mathrm{Total}}=m{˙}_{i}-m{˙}_{o}$$ 2

The current process of total water flow evaluation utilizes the total water transfer coefficient, β:

$$J_{\mathrm{Total}}=\beta \frac{j}{F}$$ 3

In proton exchange membrane (PEM) fuel cells, the well-known Nernst–Planck equation is used to model the water flow and is the summation of the electro-osmotic drag and back diffusion (if only those drivers are present); thus,

$$J_{\mathrm{Total}}=\beta \left(\frac{j}{F}\right)=n_{d}(\lambda )\frac{j}{F}-D(\lambda )\frac{\partial c}{\partial x}$$ 4

If β is positive, then electro-osmotic drag is greater than back diffusion, and the water flow will be in the direction of anode to cathode, and vice versa. In either case, knowing β does not immediately quantify either the EOD or the BD contribution, which must be indirectly calculated from the total flow. The electro-osmotic drag and back diffusion coefficients are also functions of membrane water content, as seen in Figures 1 and 2. The processing of experimental data often has underlying assumptions. For instance, if a linear trendline is forced through the origin in total water flow experiments, it implicitly assumes that the other fluid driver in this case back diffusion is negligible; thus, in the case of an EOD focused experiment,

$$J_{\mathrm{Total}}=\beta \left(\frac{j}{F}\right)=n_{d}(\lambda )\frac{j}{F}$$ 5

If forced through the origin, the impact of the other fluid drivers, if not absolutely negligible, will of course be lumped in and could either overstate or understate electro-osmotic drag, depending on the magnitude and direction of the other fluid drivers. If the other driver is in the same direction, electro-osmotic drag may be inflated and, if the other fluid driver is in the opposite direction, understated. This is the main reason the total water flow needs to be more accurately broken down into its components. A PEM fuel cell is sensitive to its heat and water management, and this requires accuracy and precision in predicting each fluid driver. The goal with the heat and water management is to keep the membrane hydrated but not too hydrated to where the cathode catalyst layer floods.

In their experiments, Ye and Wang^3^ assumed that back diffusion is negligible while indirectly measuring the electro-osmotic drag coefficient, by forcing the total water flow trendline through the origin (0 mA/cm^2^, 0 mmol/s m^2^), which will have the effect of lumping the back diffusion component to some degree into the electro-osmotic drag result. The back diffusion component of the data was never quantified, potentially leading to an inaccurate electro-osmotic drag coefficient. The linear trendline is matched to the Nernst–Planck equation; thus,

$$J_{\mathrm{Total}}=a_{o}+a_{1}j=n_{d}(\lambda )\frac{j}{F}-D(\lambda )\frac{\partial c}{\partial x}$$ 6

In the case where the current density is variable and the back diffusion is constant, the *a*_1_ term will represent electro-osmotic drag and the *a*_o_ term will represent back diffusion. In a Nafion membrane, however, the electro-osmotic drag coefficient and back diffusion coefficient are dependent on the hydration state and are not constant. Therefore, the linear fit does not account for any changes in the electro-osmotic drag coefficient over a sizable range. Yan et al.^19^ performed an experiment on a 25 cm^2^ active fuel cell to see how operating conditions impact the fuel cell performance. In one of the experiments, the stoichiometric ratio of hydrogen and the inlet relative humidity was fixed and the current density was varied, with the initial results expressed in terms of the total water transfer coefficient. With the use of eq 2, the total water flux across the membrane can be expressed with respect to current density, as shown in Figure 4.

**Figure 4:**
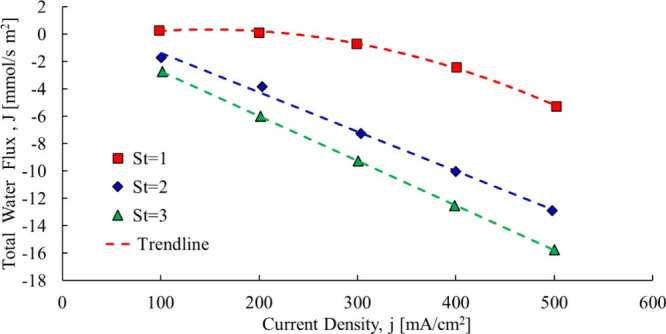
Total water transfer coefficient data—Yan et al.^19^

Two of the three cases can fit to a linear trendline (at least apparently), with a clearly nonlinear one when the hydrogen stoichiometric ratio is unity. Based on the Nernst–Planck eq 4, the total water flow should be linear in all cases. Note that the anode was fully humidified and the cathode was partially humidified, and the experiment was conducted in a nonaqueous solution performed with water vapor. If the trend is not linear for St = 1, an argument can be made that there may be minor nonlinearities in the other data sets even if not apparent with the naked eye.

Springer et al.^20^ performed experiments using a 50 cm^2^ active area fuel cell and varied the operating conditions. Similar to Yan et al.,^19^ they fixed the stoichiometry of the hydrogen and kept the inlet relative humidity constant while varying the current density, the initial results expressed in terms of the total water transfer coefficient. With the use of eq 2, the total water flux across the membrane can be expressed with respect to current density as follows:

Referring to Figure 5, the total water flow across the membrane in all cases is fitted to a second-order polynomial trendline, as here it is apparent that there is no linearity in any of the data sets. Since both the anode and cathode were fully saturated, liquid water will be produced by the fuel cell. It seems then that as the relative humidity increases on either (the anode or cathode), the total water flow deviates from the linear. With that as a clue, an experiment was selected where the total water flow was measured with liquid on both sides of the membrane. The Barragan et al.^21^ total water flow data in an aqueous solution is shown in Figure 6.

**Figure 5:**
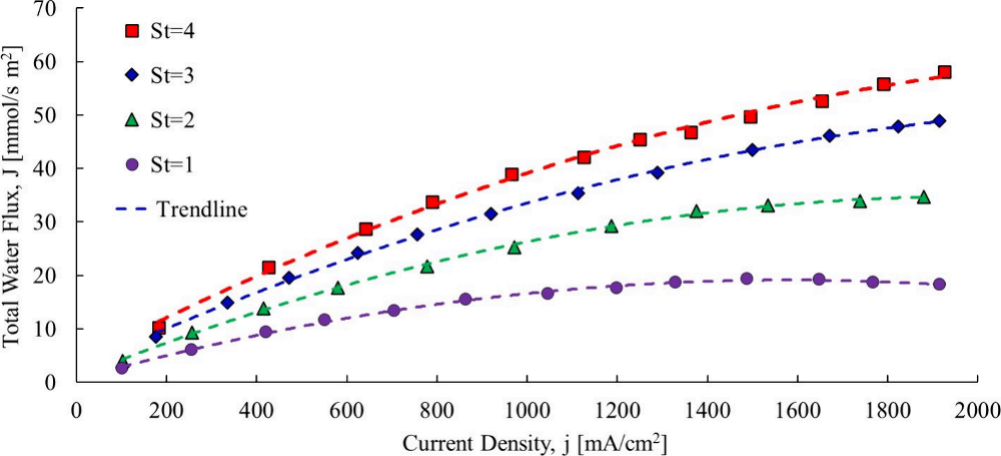
Total water flow transfer coefficient data of Springer et al.^20^

**Figure 6:**
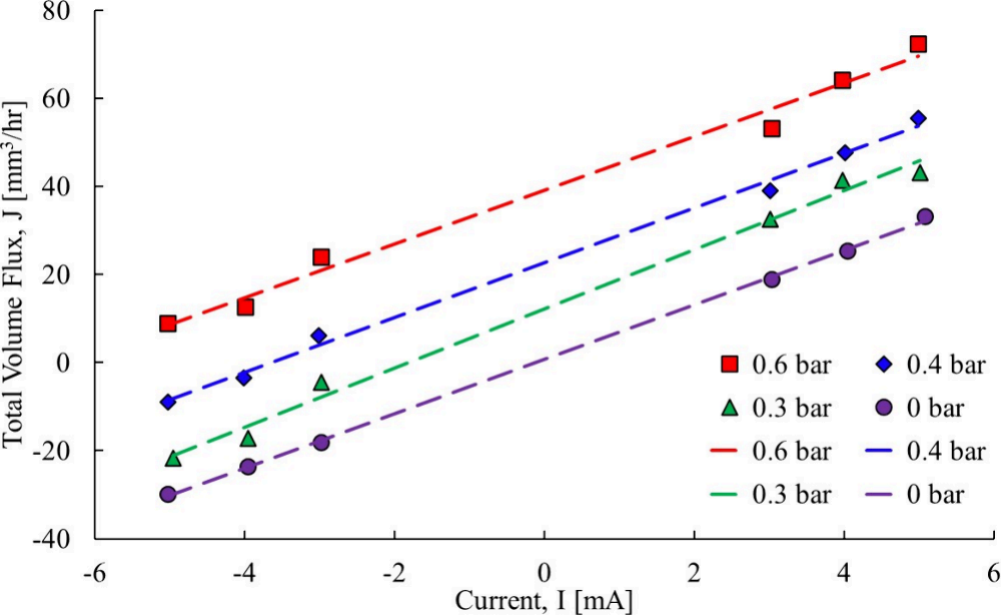
Total water flow transfer across a Nafion 117 membrane.^21^

The total water flow across the membrane in Figure 6 appears to vary linearly with the current for all of the cases. As the pressure component increases, the linear trendlines move further away from the origin. The only case that was close to the origin is the 0 bar data set. Here, the trendlines do not go through the origin in any of the cases, indicating that electro-osmotic drag is not the only fluid driver. Ye and Wang^3^ stated that the membrane was sufficiently thick, and back diffusion can be neglected, but this assumption made without justification, and an experiment was not performed with several membrane thicknesses to determine at which point back diffusion was indeed negligible. That is a shortcoming that the current research will address.

Adachi^22^ studied how membrane thickness impacts the total water flow across the membrane when the anode is dry and the cathode fully saturated. The study looking at total water flow across the membrane changes with respect to current density, as shown in Figure 7.

**Figure 7:**
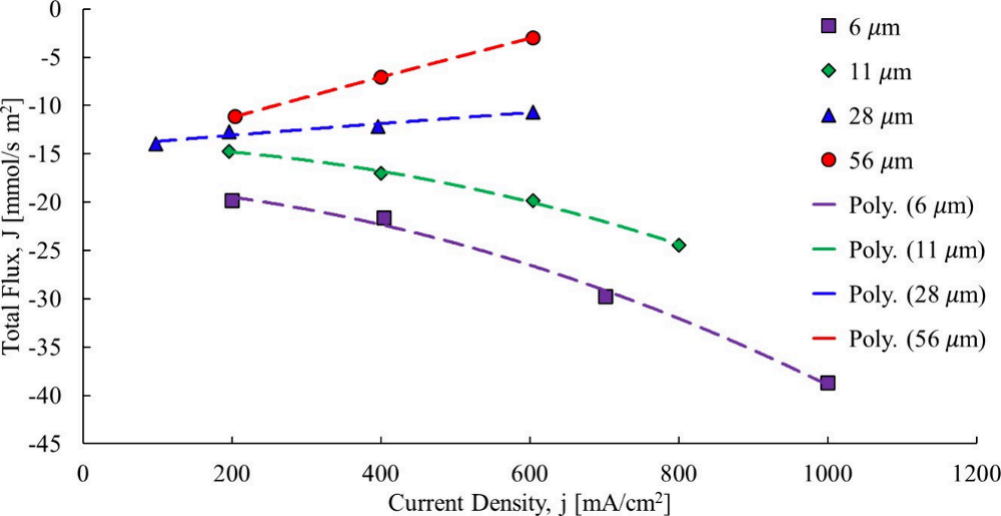
Total water flow transfer fuel cell membrane.^22^

In cases where the membrane is at thicknesses of 6 and 11 μm, the data trends are clearly nonlinear indicating the presence of another driver, in this case back diffusion. Thicker membranes, however, reduce back diffusion because they decrease the concentration gradient. Referring to the 28 and 56 μm data sets in Figure 7, even though they appear linear, the limited range contributes to the obfuscation of perhaps slight nonlinearities still present albeit far less pronounced.

Bieshevel and Dykstra^23^ noted that electro-osmotic experiments contain volume flow from two components: flow of hydrated ions and flow of water. It therefore requires two experiments to compute the electro-osmotic drag coefficient: one run with variable osmotic pressure to determine the flow due to that pressure difference and the second with electro-osmotic-driven flow. The difference between the two yields the electro-osmotic flow component. Thus, developing a method for EOD and BD driver measurement involves addressing several challenges, as current research depends upon the validity of past studies. Ye and Wang^3^ isolated electro-osmotic drag with one experiment but did not obtain just the electro-osmotic drag portion of the total water flow, instead reporting the total water flow.

The Ye and Wang^3^ total water flow experiments were conducted with a hydrogen pump to determine the electro-osmotic drag coefficient. They did not perform an experiment where the total water flow was measured without a hydrogen pump subtracting the osmotic pressure to determine the electro-osmotic drag coefficient. Instead, they relied on the slope of the trendline to compute it. This implicitly assumes that the back diffusion is negligible. In fact, Ye and Wang^3^ stated that the membrane was sufficiently thick, and the back diffusion can be neglected and again was done so by forcing the trendline through the origin. However, this has the effect of assuming that all the fluid flow across the membrane is from electro-osmotic drag, and the impact of back diffusion is zero. It will be shown here that that is not the case. In addition to neglecting back diffusion, the linear trendline used by Ye and Wang^3^ was fitting to all data sets rather than separating out individual relative humidity sets. According to Kusoglu and Weber,^1^ the electro-osmotic drag coefficient and back diffusion coefficient are impacted by water content of the membrane, and the existing approach is agnostic to that. The objective of the research here is thus to reexamine total water flow data and extract an electro-osmotic drag and back diffusion coefficient and to determine if the back diffusion component was truly negligible. Figure 8 shows the Ye and Wang^3^ data, still forced through the origin, but here separated by relative humidity, showing slight differences because the electro-osmotic drag coefficient changes with hydration state.

**Figure 8:**
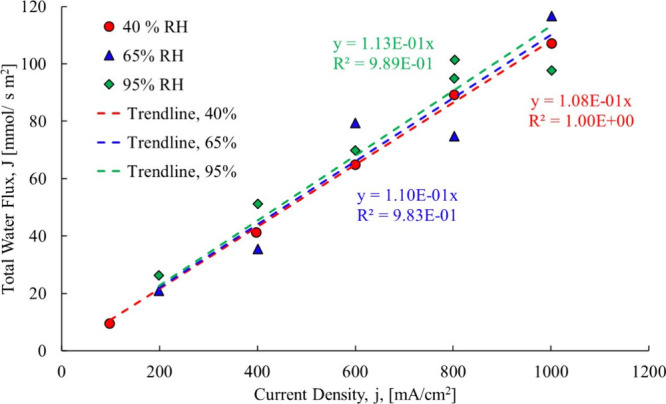
Ye and Wang^3^ total water flow data, Gore-Select Membrane—case 1.

Again, they fit a trendline through all data points and by forcing it through the origin implicitly assume that no other drivers are present. The individual slopes of the trendlines, however, show that the relative humidity impacts the electro-osmotic drag coefficient. Yand Wang^3^ noted, at least qualitatively, that their membrane was thick enough where back diffusion could be neglected but offered no justification. They performed a second experiment with the same relative humidity levels but with a different membrane (Nafion versus Gore-Select in their first experiment) with the results shown in Figure 9, but again with trendlines separated by humidity levels (still forced through the origin as per the former research):

**Figure 9:**
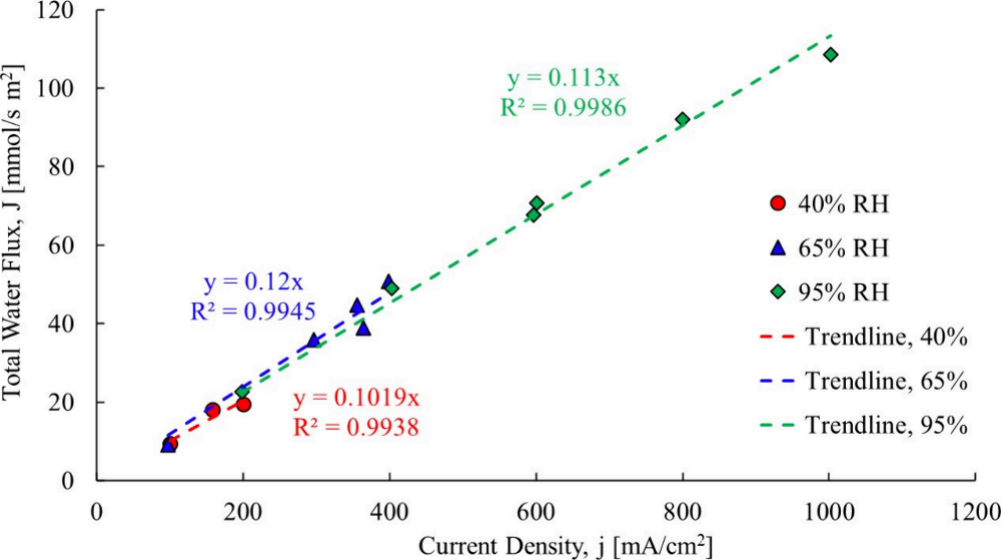
Total water flow forced through the origin—Nafion Case 2.^3^

Again, there are differences between the trendlines due to the different hydration states of the membrane. Each of the relative humidity trendlines has a different slope indicating that the electro-osmotic drag coefficient will change with relative humidity. It is pointed out again here that Bieshevel and Dykstra^23^ indicated that electro-osmotic drag is not the only fluid driver present in any EOD experiment and that multiple experiments have to be performed to get at its unique contribution. With the complexities of isolating and thus accurately accounting for the various fluid drivers present, the goal here is to lay out a method to do just that identify and accurately separate total water flow into components for each of the fluid drivers with one experiment.

## Theory

As shown in Figures 1 and 2, the EOD and BD coefficients are dependent on the hydration state of the membrane, which varies (possibly substantially) depending on the empirical model chosen. In addition to the scatter, inconsistencies in results can also arise due to the presence of other minor fluid drivers during the experiment—ones that are easily considered to be negligible. There are four primary drivers of fluid flow: temperature gradient, concentration gradient, electropotential, and pressure difference, as shown in Figure 10.

**Figure 10:**
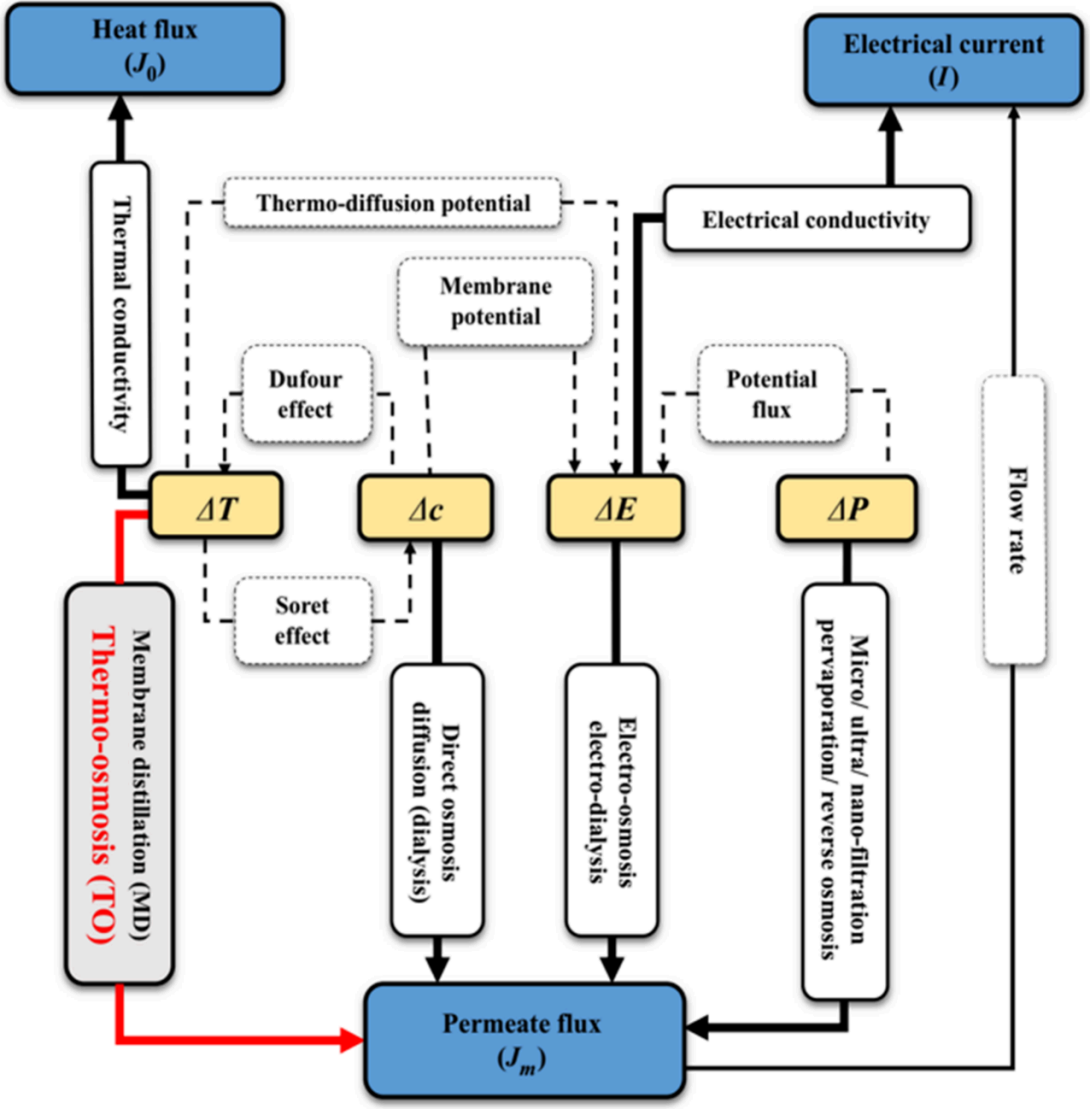
Fluid flow drivers.^24^

Secondary effects, such as the combination of voltage gradient and concentration gradient yielding membrane potential, can also influence results.^24^ It is not clear many times whether other fluid drivers are indeed negligible. There are two fluid drivers from the thermal gradient: thermal osmosis and membrane distillation, the latter possibly occurring under isothermal conditions when liquid water is present on at least one side of the membrane (also known as osmotic distillation), but these drivers are beyond the scope of this current research. The research here involves coupling the theoretical model (Nernst–Planck) with experimental results to solve for the electro-osmotic drag and back diffusion coefficients at each data point.

The first step is to establish the theoretical model for total water flow across the membrane, and the second curve is fitting the total water flow across the membrane with respect to the current density. The total water flow across a membrane is modeled with the well-known Nernst–Planck equation:

$$J_{\mathrm{Total}}=n_{d}(\lambda )\frac{j}{F}-\frac{\rho }{EW}D_{\lambda }\frac{d\lambda }{dx}$$ 7

Referring to eq 7, the concentration gradient along with the electro-osmotic drag drives total water flow. If the derivative of the Nernst–Planck equation is taken with respect to current, only the electro-osmotic drag coefficient will remain; thus,

$$\frac{dJ_{\mathrm{Total}}}{dj}=\frac{n_{d}(\lambda )}{F}$$ 8

On the other hand, if the derivative of the Nernst–Planck equation is taken with the respect to the concentration gradient, only the Fickian diffusion coefficient will remain:

$$\frac{dJ_{\mathrm{Total}}}{d\left(\frac{d\lambda }{dx}\right)}=-\frac{\rho }{EW}D_{\lambda }$$ 9

Obtaining a trendline to experimental data in the case where only two fluid drivers are present should correspond to a linear trendline: thus,

$$J_{\mathrm{Total}}=a_{o}+a_{1}j$$ 10

The linear fit of the data contains two components: the *a*_o_ term that would contain back diffusion and other fluid drivers (this being so because the trendline here would not be expected to go through the origin), and the *a*_1_ term that would contain only the electro-osmotic drag term. It is noted here that if the function is forced through the origin, the *a*_o_ term quantifying other fluid drivers would be zero *implicitly neglecting* other drivers and resulting in improperly skewing EOD results. If the derivative of eq 10 is taken with respect to current, only a constant would remain:

$$\frac{dJ_{\mathrm{Total}}}{dj}=a_{1}$$ 11

To solve for the electro-osmotic drag coefficient, eq 11 is set equal to eq 8 resulting in

$$\frac{n_{d}(\lambda )}{F}=a_{1}$$ 12

In the case of the linear fit, it also assumes that the electro-osmotic drag coefficient is constant and does not change with respect to current density. However, as the water flow changes across the membrane, the hydration of the membrane will change, which will in turn change the electro-osmotic drag coefficient. The hydration state of the membrane also impacts the back diffusion coefficient. In a PEM fuel cell, water is generated on the cathode side, and as a result, liquid water can form and introduce another transport mechanism. Ingarra et al.^25^ noted that water vapor and liquid water will flow differently through the membrane of the fuel cell, and as a result, the single-phase and two-phase water flow must be isolated from one another to obtain a good trendline fit, as shown in Figure 11a,b.

**Figure 11:**
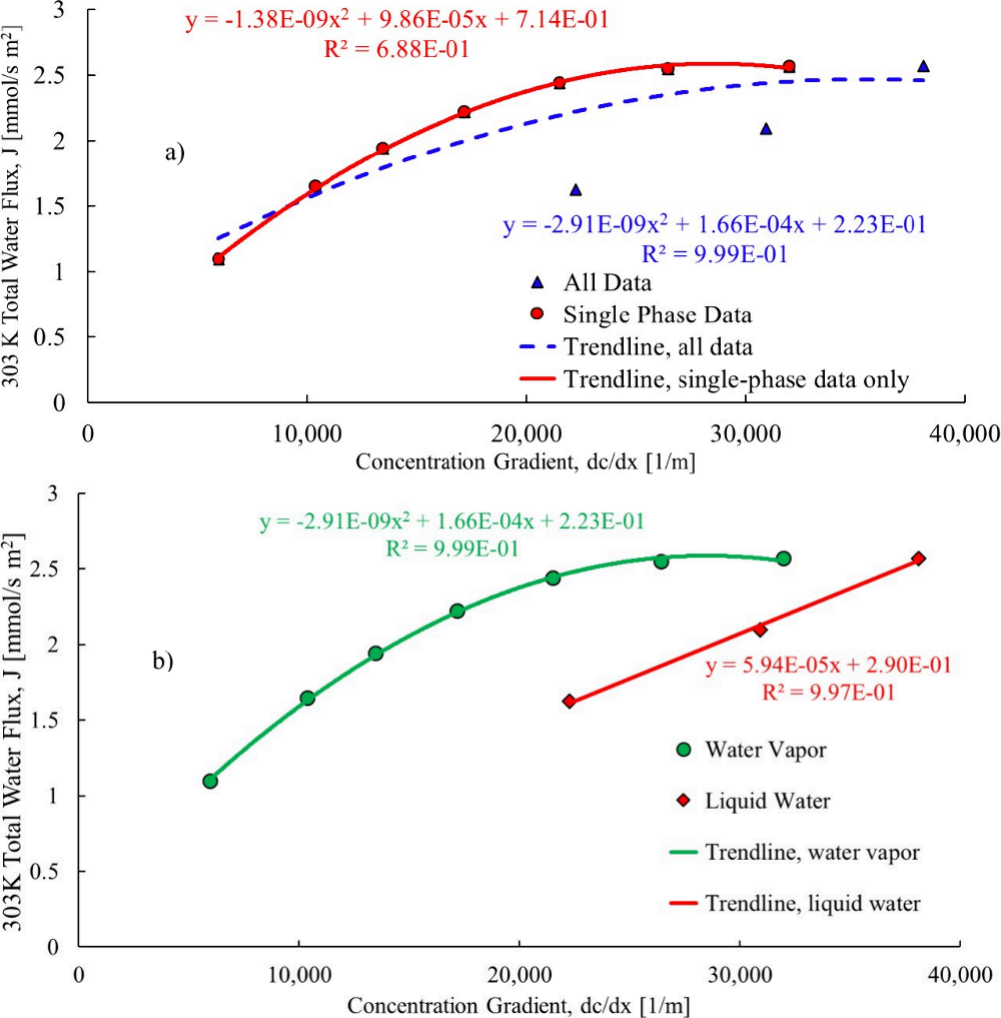
Total water flow being isolated in single- and two-phase.^25^

Figure 11a shows a plot of all data and a good correlation was not achieved, and the isolated single phase and an improved correlation was achieved. Figure 11b shows the same data with the single-phase and two-phase plotted separately with good correlations achieved in both cases. The results show that total water flow across the membrane varies linearly with liquid water present but varies nonlinearly when water vapor is present. If the total water flow from Yan et al.^19^ and Springer et al.^20^ are examined further, then the total water flow does not vary linearly with current density in some of the cases. This is the likely result of other fluid drivers present, such as the two-phase transport mechanism membrane distillation or osmotic distillation as well as the water formation at the cathode. Thus, with possible nonlinearities present, the total water flow data is fitted to a second-order polynomial:

$$J_{\mathrm{Total}}=a_{o}+a_{1}j+a_{2}j^{2}$$ 13

Moreover, the derivative yields

$$\frac{dJ}{dj}=a_{1}+2a_{2}j$$ 14

The experiment data in eq 14 is then set equal to the derivative of the Nernst–Planck eq 8; thus,

$$\frac{n_{d}(\lambda )}{F}=a_{1}+2a_{2}j$$ 15

With the electro-osmotic drag coefficient computed using eq 15 or 12, the electro-osmotic drag portion of the flow can be computed as follows:

$$J_{\mathrm{EOD}}=\frac{n_{d}(\lambda )}{F}j$$ 16

With the EOD portion computed, the back diffusion portion of the total water flow can be computed as well using eq 4, and the magnitudes of these two fluid drivers are compared:

$$R_{\mathrm{EOD}}=\frac{\left|J_{\mathrm{EOD}}\right|}{\left|J_{\mathrm{EOD}}|+|J_{\mathrm{BD}}\right|}$$ 17

$$R_{\mathrm{BD}}=\frac{\left|J_{\mathrm{BD}}\right|}{\left|J_{\mathrm{EOD}}|+|J_{\mathrm{BD}}\right|}$$ 18

It should be noted that the Nernst–Planck equation does not recognize temperature-driven flow; there are two forms of temperature-driven flow, thermal osmosis and membrane distillation. Thermal osmosis is a single-phase fluid transport that is driven by the thermal gradient, and it could drive fluid in the hot to cold direction or cold to hot direction; it must be investigated further to incorporate it into fuel cell models

The second form of thermal osmosis is membrane distillation, and this will occur only when liquid water is present with a hydrophobic membrane like PTFE. In this case, the water must evaporate to pass through the membrane and condense on the other side. The water flow from membrane distillation will occur in the hot to cold direction. There is also an isothermal version of membrane distillation called osmotic distillation. In this case, the water transport will go in the direction of liquid to vapor and it is driven by the vapor pressure differences. All of these effects are not captured in the Nernst–Planck equation, and they must be understood.

## Results

The first step utilizing the current proposed process is to refit the Ye and Wang^3^ experimental data to where the total water flow is neither forced through the origin nor with a linear fit, due to the shortcomings of both as explained in the Theory section of this paper. The resulting trendlines are shown in Figure 12.

**Figure 12:**
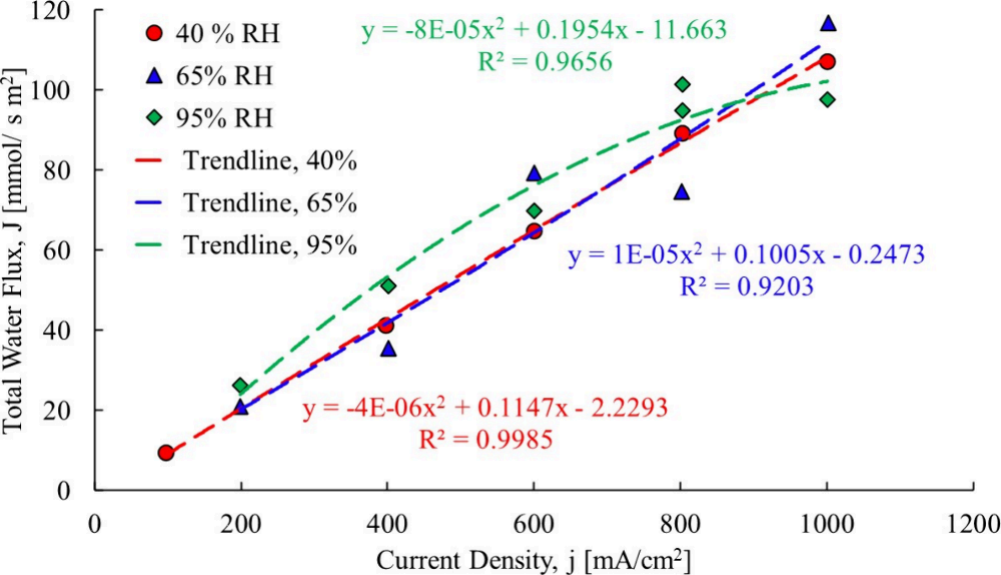
Ye and Wang^3^ total water flow—case 1.

The first membrane examined is a Gore-Select membrane, which contains hydrophobic regions that would be impacted when liquid water is present. A polynomial fit was used for all three cases, the 40% RH and 65% RH and 95% RH. For the linear fit, eqs 10–12 are used, and for the second-order polynomial data fit (used to capture the changing of the electro-osmotic drag coefficient at each data point), eqs 13–15 were used to compute the electro-osmotic drag coefficient. With the proposed method, the electro-osmotic coefficient is determined at each data point, with the results shown in Figure 13.

**Figure 13:**
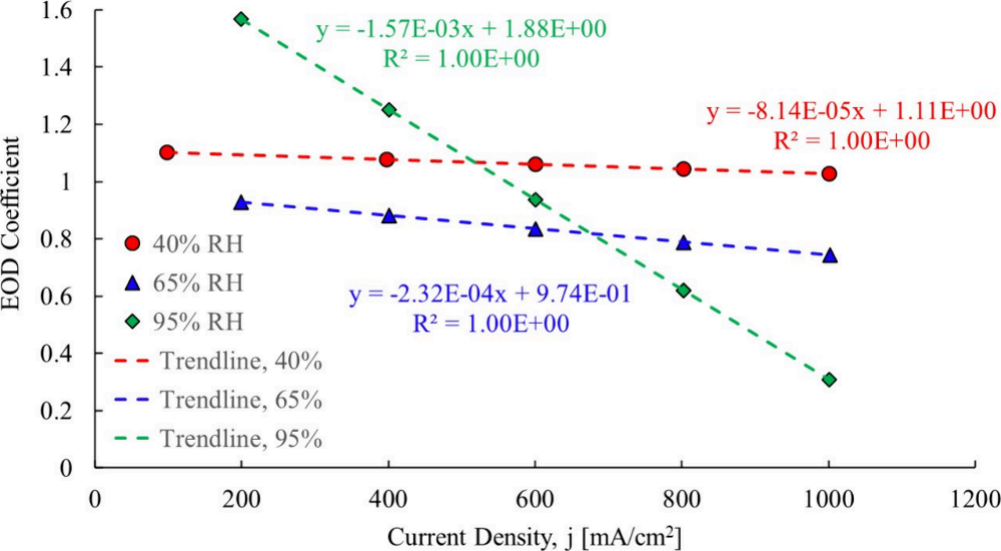
Ye and Wang calculated electro-osmotic drag coefficient—case 1 Gore-Select 200 μm.^3^

The original data analysis produced an electro-osmotic coefficient of 1.07. By isolating each relative humidity set, it was found that the relative humidity impacts the electro-osmotic drag coefficient: The 40% RH data produce an electro-osmotic drag coefficient that is a weak function of current density starting off at 1.10 and decreasing to 1.03. For the 65% RH case, the data show it also as a function of current density ranging from 0.92 to 0.74. The 95% RH scenario shows the electro-osmotic coefficient decreasing from 1.58 to 0.3, at 95% RH. Referring again to Figure 13, the 95% RH case in particular clearly shows electro-osmotic drag as a strong function of current density.

The electro-osmotic drag coefficient may not be constant due to changes in the hydration state of the membrane as well as to the liquid water on the cathode, which is possible at higher relative humidity. Adachi^22^ noted that when a membrane has liquid water on one side and vapor on the other, the direction of the water flow will be in the direction of liquid to vapor. Ingarra et al.^26^ noted the liquid to vapor transport phenomena is either membrane distillation or osmotic distillation and that the Nernst–Planck equation does not cover both terms.

The electro-osmotic drag and back diffusion portions of the total water flow are computed with eqs 17 and 18. The calculated electro-osmotic drag and back diffusion portions of the total water flow for the three humidity levels, respectively, are shown in Figure 14.

In the 40% RH scenario, the back diffusion portion of the total water flow ranges from 1.1 to 10%. The percentage of back diffusion is not constant; it varies with current density. In the 65% RH case, the back diffusion portion ranges from 1.3 to 18.7%. Small but certainly not strictly negligible.

In the 95% RH case, the back diffusion portion of the total water flow ranges from 1.7 to 25.3%. The electro-osmotic drag coefficient decreases with current density, and at the higher current density, the back diffusion dominates; this is due to the “Schroder Paradox”, otherwise known as membrane distillation/osmotic distillation.

The main concern in the prior research is that the original electro-osmotic drag reported was a total water flow assuming no other drivers. As shown here, the 40% RH scenario produced an electro-osmotic drag coefficient of 1.06 and ranges from 1.10 to 1.03. The reason for the changes is that they are a function of the hydration state of the membrane, which is not itself a constant with current density. The original trendline was in error to some extent assuming all the fluid flow is due to electro-osmotic drag alone.

**Figure 14:**
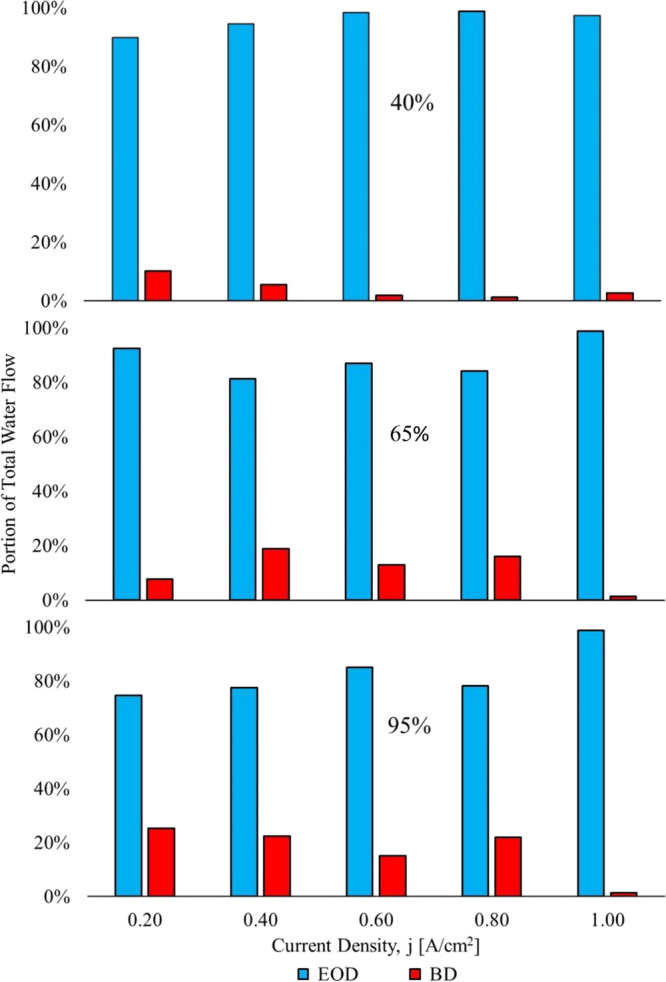
Total water flow breakdown case 1.^3^

In the 65% RH scenario, the calculated electro-osmotic drag coefficient ranges from 0.92 to 0.74 while the original value reported was 1.10, the difference depending on the current density. In the 95% RH scenario, the total water flow was fitted with a second-order polynomial, and as a result the electro-osmotic drag (the derivative of the second-order function) is going to vary linearly with current density. At high relative humidity, there is a high probability of liquid water condensing on the cathode. The difference between the new and original electro-osmotic drag models is shown Figure 15.

**Figure 15:**
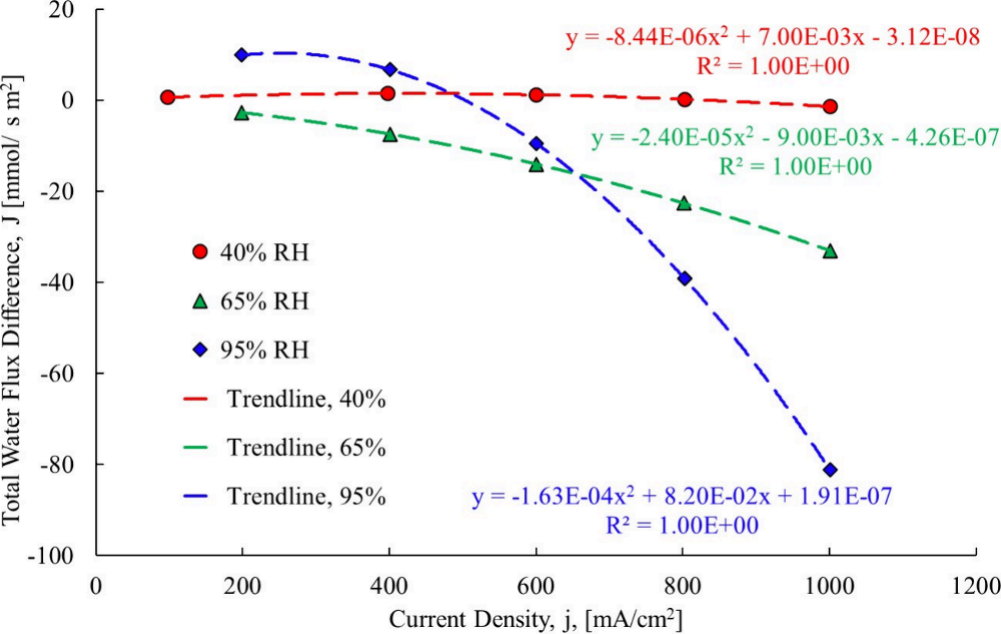
Difference between new electro-osmotic and original case 1.^3^

One of the reasons for the difference between the prior research and this one is the accounting for back diffusion, which means that the original electro-osmotic drag coefficient has a small back diffusion factor “baked into” the result. The difference between the prediction for the 40% starts off positive and then goes negative, indicating that the electro-osmotic drag was initially positive meaning more water was transported via electro-osmotic drag, but as the current density increased, the difference became negative meaning that less water was transported via electro-osmotic drag. In the 65% RH scenario, the difference starts off negative and remains so, which means the prior model will overstate the electro-osmotic drag and less water will be present at the cathode. The negative difference is due to the calculated electro-osmotic drag coefficient being less than the original reported result. The 95% case shows that the models diverge more: As the current density increases, the difference becomes more negative, showing that less water from electro-osmotic drag is passing through the membrane and could result in overhumidification of the cathode if the humidifier was sized for the original electro-osmotic drag coefficient.

The second Ye and Wang^3^ data set is examined following the same procedure. The Nafion membrane thickness is 250 μm, and its structure contains hydrophobic and hydrophilic regions, the hydrophobic regions inducing membrane distillation and/or osmotic distillation when liquid water is present. A second-order polynomial fit was used for the 40% RH, 65%, and 95% RH cases. Each data run was isolated and plotted, as shown in Figure 16.

**Figure 16:**
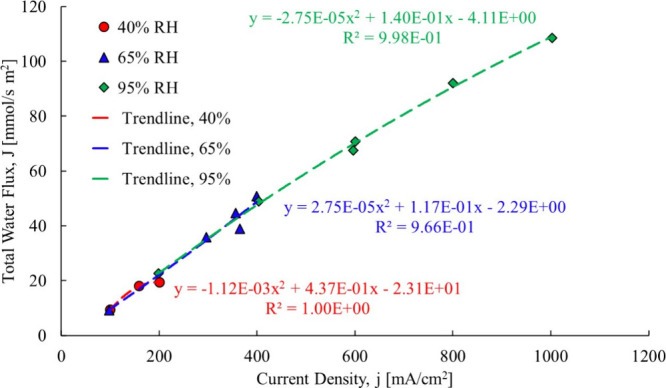
Ye and Wang^3^ reprocessed total water flow—case 2.

For the second-order polynomial data fit, eqs 13–15 were again used to compute the electro-osmotic drag coefficient. Again, with this proposed method, the electro-osmotic coefficient is determined at each data point with the results shown in Figure 17:

**Figure 17:**
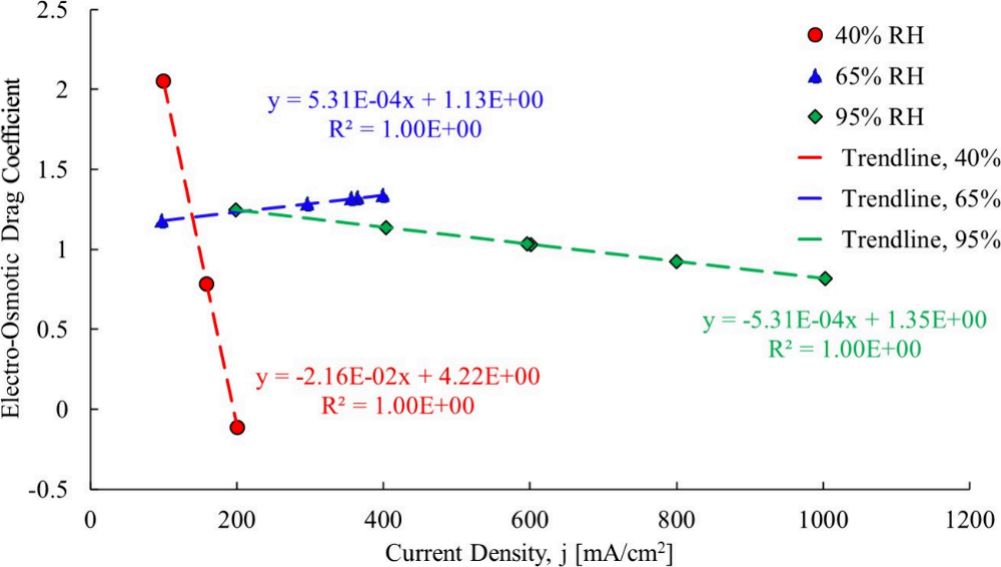
Ye and Wang^3^ calculated electro-osmotic drag coefficient—case 2.

In the 40% RH case, the electro-osmotic drag decreases with current density, starting at 2.05 and turning negative, due to its changing with the hydration state of the membrane. The limited data points are a result of the higher resistance in the membrane. In the 65% RH case, the electro-osmotic drag coefficient starts at 1.18 and increases to 1.34, the change due the changes in the hydration state of the membrane. All results show the electro-osmotic drag coefficient as a function of current density, unlike the original research.

In the 40% RH case, the back diffusion portion of the total water flow was as high as 90% and in the 65 and 95% RH cases up to 20%. This signifies that the back diffusion is not only present in the experimental data but is substantial. The original results reported by Ye and Wang^3^ had this BD effect in the EOD results, which renders the latter skewed. The composition of the total water flow is shown in Figure 18.

**Figure 18:**
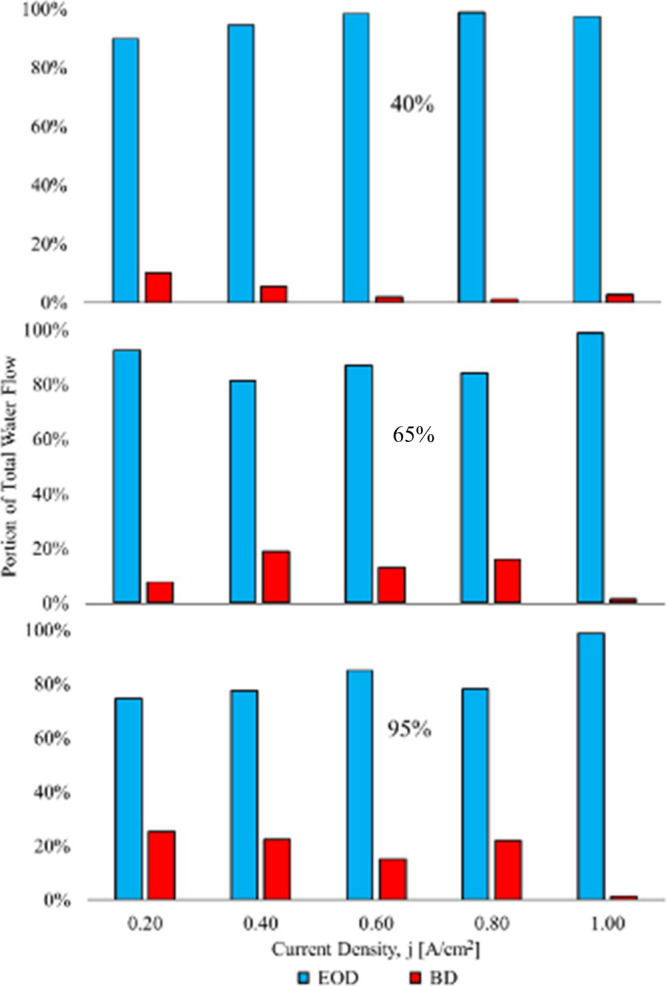
Composition of the total water flow.^3^

In the PEM fuel cell, there are two currents generated by the oxidation reaction of hydrogen: the first being the electrical current, which produces electrical power that can be measured, and the second being the hydrogen ion, which crosses the membrane via electro-osmotic drag. As the current density increases, the voltage gradient across the membrane decreases, lowering the voltage gradient that could, in turn, lower the electro-osmotic drag coefficient. This would seem to be very important insight into the scope of being able to control fuel cell functionality to optimize performance.

The difference in the electro-osmotic drag coefficient will yield a different amount of water on the cathode. If the cathode was humidified based on the existing model, the cathode will be overhumidified and produce liquid, which is undesirable. The differences between the new and original models is shown in Figure 19.

**Figure 19:**
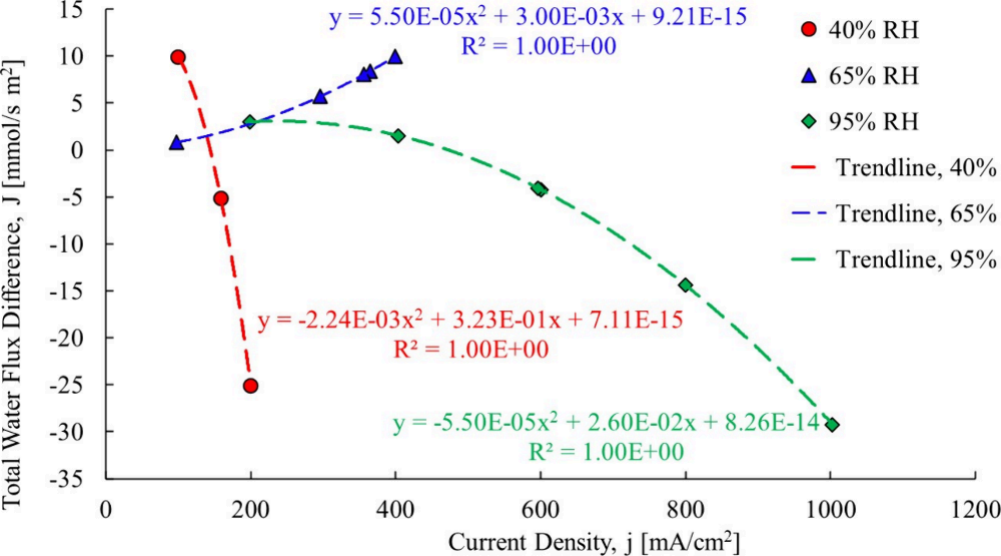
Difference between proposed model and original electro-osmotic drag coefficient.^3^

The difference in the 40% RH case starts off positive at a low current density and turns negative at larger. When the results are positive, the new model indicates that more water will be transported compared with the original model. When the difference is negative, the new model shows that less water is being moved than originally predicted. The 95% case shows a major divergence between the two models. The difference in all cases stems from the fact that the new model does not have the back diffusion component lumped into the results like the former research appeared to do. Another reason for the variation is from the change in the membrane hydration state with increasing current density. Ye and Wang^3^ conducted an electro-osmotic drag experiment with a 25 μm Gore-Select membrane, the thinner membrane promoting more back diffusion. The total water is shown in Figure 20:

**Figure 20:**
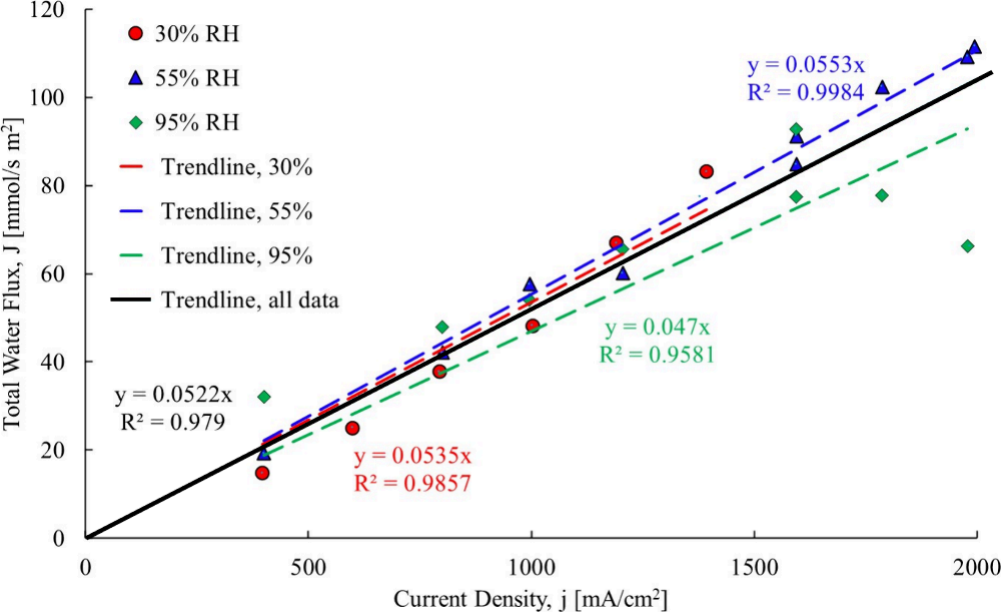
Total water flux data trendline forced through the origin, Gore-Select 25 μm membrane.^3^

In this figure, the original linear trendline was forced through the origin and included all the data points as shown. Here, separate trendlines were used for the various data sets run at different RH levels. The slope of each data trendline is different, and this indicates that the electro-osmotic drag is a function of the relative humidity. The data fits here do neglect the back diffusion component and that the electro-osmotic drag coefficient is not a function of current density.

Again, it is noted here that forcing these linear trendlines through the origin as has been done in many prior research efforts assumes (implicitly) that electro-osmotic drag is the only fluid driver. However, without quantifying back diffusion, it is an unvalidated assumption. In addition, assuming linearity can also conceal additional effects. In this light, total water flow data was best fit to a second-order polynomial to capture the variation on the electro-osmotic drag and back diffusion coefficient as shown in Figure 21.

**Figure 21:**
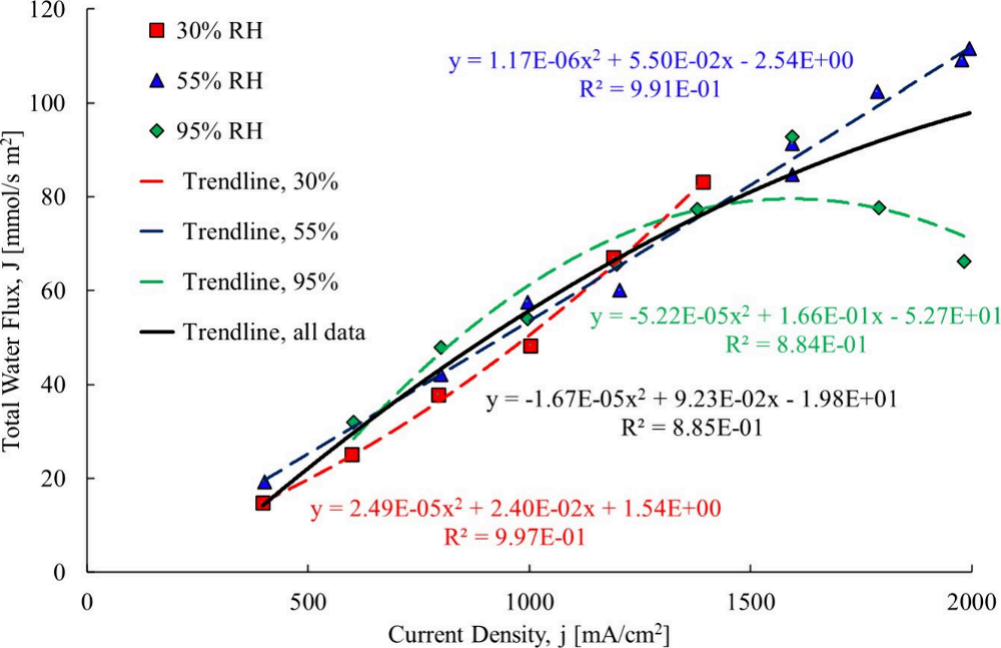
Total water flux data trendline—25 μm membrane.^3^

The total water flow data was fit to a second-order polynomial trendline for all RH cases. The 95% RH case is the only one that showed a decrease in total water flow above a current density of approximately 1500 mA/cm^2^. Due to this unique decrease, the data were further broken down into two regions: a single- and two-phase. This was done because at high relative humidity, liquid water could be liquid water present. The two regions are shown in Figure 22.

**Figure 22:**
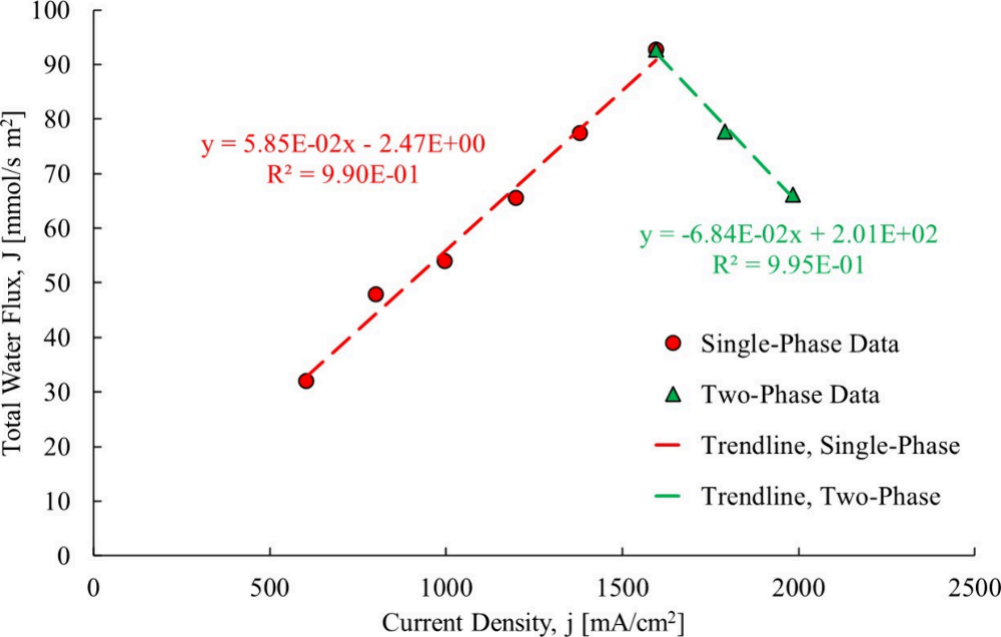
95% RH data broken down into two regions—25 μm membrane.^3^

The regions were separated at the current density where there appeared to be a dramatic change in the data from increasing to decreasing, around 1500 mA/cm^2^. The trendline in the single-phase region shows a constant electro-osmotic drag coefficient as the water flow is increasing up to 1500 mA/cm^2^. Above this current density, it becomes negative, indicating that the direction changes from anode to cathode to cathode to anode. This is due to the presence of liquid water that introduces membrane/osmotic distillation, which will move it in the direction of liquid to vapor.

It is noteworthy here that this additional information is being “mined” by *not* fitting the experimental data to a linear function. Past linear data fits indeed concealed this major change in fluid flow direction, and it is one of the improvements detailed in this current research. With this insight, the electro-osmotic drag coefficient is calculated using the current method with the results shown in Figure 23.

**Figure 23:**
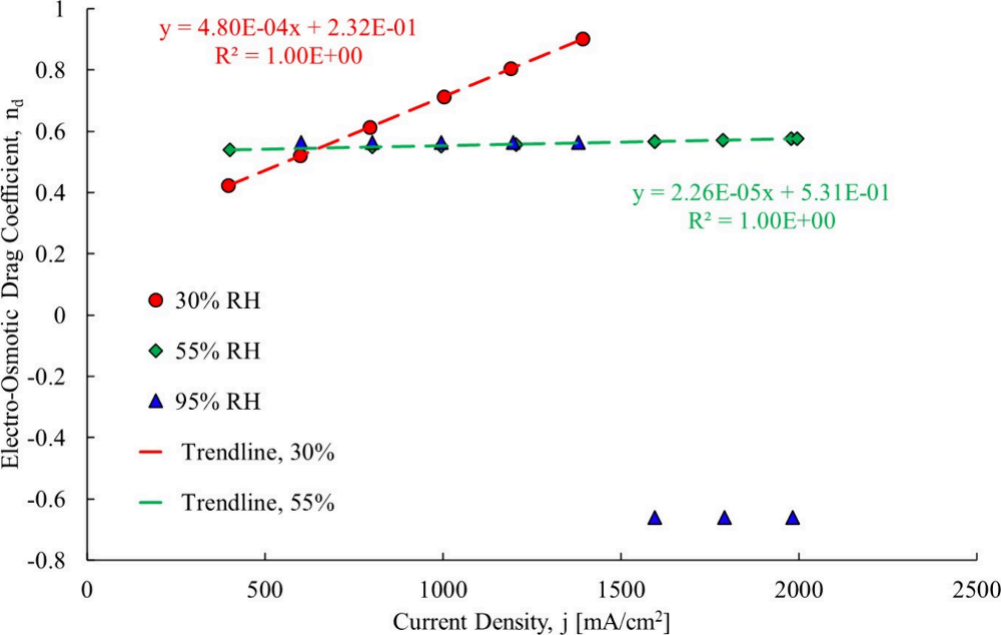
Computation of the electro-osmotic drag coefficient.^3^

The electro-osmotic drag coefficient for the 30% RH case ranged from 0.42 to 0.9, and it is increasing with current density. The original research reported a constant value of 0.51. The change in the electro-osmotic drag coefficient is due to the changing hydration state of the membrane with an increasing current density. The back diffusion is working against electro-osmotic drag, as the Nernst–Planck equation shows. The electro-osmotic drag coefficient for the 55% RH case ranges from 0.53 to 0.57 over a large range of current densities. For the 95% case at low current density, the electro-osmotic drag coefficient is around 0.56, but when the current density is higher, the electro-osmotic drag coefficient becomes −0.66 indicating that the direction shifts to from cathode to anode, while the back diffusion goes continues from anode to the cathode. If liquid water is present on the cathode, then osmotic distillation could occur and drive the water flow in the direction of liquid to vapor. With all of the above data mining, the total water flow composition is determined using the current research with the results shown in Figure 24.

**Figure 24:**
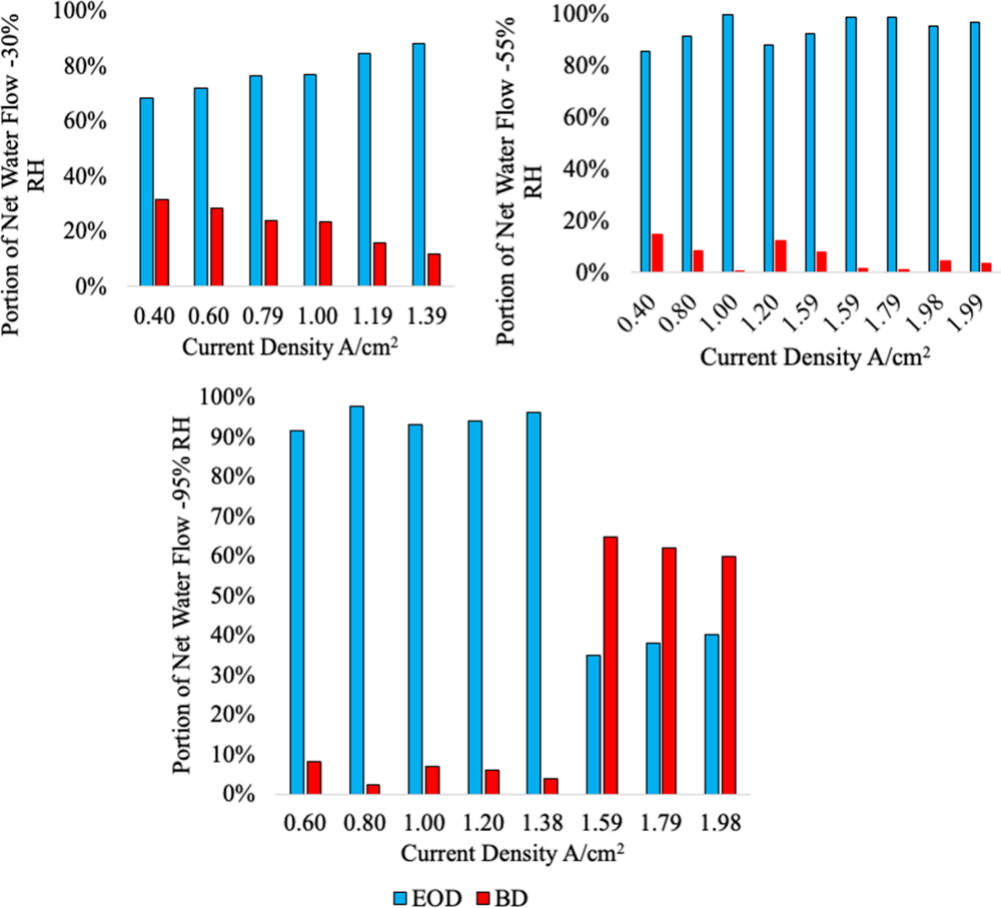
Composition of total water flow for 25 μm.^3^

In the 30% RH case, the back diffusion component of the total water flow ranges from 11.85 to 31.5%, the thinner membrane promoting more back diffusion. These are certainly non-negligible numbers as claimed in the original prior research. In the 55% RH case, the back diffusion portion of the total water flow ranges from 1 to 14.5%, so some of the data points contain negligible back diffusion and others do not. In the 95% RH case, the back diffusion ranges from 2.20 to 65%. The back diffusion in the single-phase region ranges from 2.2 to 8.3% and in the two-phase region from 59.8 to 65%. In the two-phase region, osmotic distillation can occur, which will drive the water flow in the direction of liquid to vapor, which would go against electro-osmotic drag. The difference of the electro-osmotic flow will impact the water balance in the fuel cell. The difference between the former and current models is shown in Figure 25.

**Figure 25:**
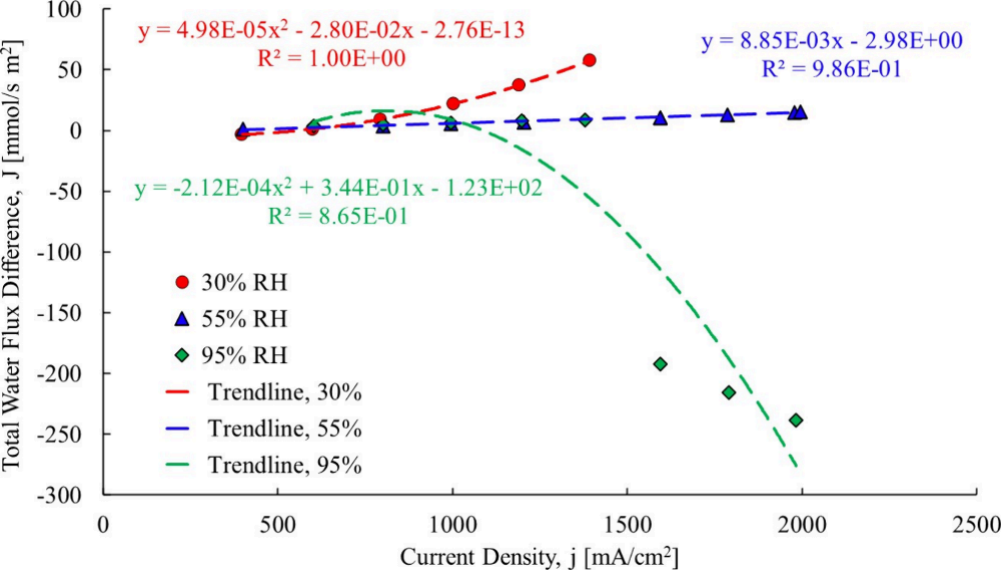
Difference between two electro-osmotic drag models.^3^

In the 30 and 55% RH cases, the electro-osmotic drag model here shows that more water is being transported via electro-osmotic drag compared to the original results because the current model extracted the back diffusion component from the total water flow. As a result, more water was transported to the cathode, which could have led to liquid water forming on the cathode. In the 95% RH case, the current model shows that more water is being transported up to 1500 mA/cm^2^. When the current density is greater than 1500 mA/cm^2^, the electro-osmotic drag is in the cathode to anode direction due to osmotic distillation coming into play. This is the main reason the electro-osmotic drag portion is decreasing.

To break down the total water flow to obtain back diffusion and thermal osmosis transport coefficients, more experimental data is needed as well as additional measurements. To obtain the thermal osmosis transport coefficient, the temperature of the anode and cathode catalyst layers is needed to compute the thermal gradient. The temperature measurements are also needed to compute the saturation pressure of air. The vapor pressure of the water also needs to be computed in the catalyst layers to compute the thermal gradient. The phase of water needed to be noted at each data point because osmotic or membrane distillation will occur once the liquid water is present.

## Conclusions

When the total water flow across the membrane is indirectly measured, it is a result of all of the simultaneous fluid drivers present. In prior experiments, the total water flow was broken down into its electro-osmotic drag and back diffusion components (assuming that those were the only drivers present). However, attempting to do so came with assumptions lacking justification. One of the common assumptions in total water flow experiments is implied by forcing the data trendline through the origin, which assumes no other fluid drivers are present. If indeed other minor fluid drivers are at play, then this will have the effect of lumping those minor drivers into the indirectly measured variable. In addition, a linear trendline is used because it follows from the Nernst–Planck equation. However, utilizing linear trendlines also assumes that the electro-osmotic coefficient is constant and can conceal other effects as has been shown here. Indeed, nonlinear behavior has been seen in total water flow data. As the relative humidity increases, the total water flow trendline becomes less linear and resembles a higher-order polynomial (second-order polynomials suffice). Also, in the PEM fuel cell membranes, the electro-osmotic drag and back diffusion coefficient depend on the membrane hydration state.

The modeling of the total water flow across the fuel cell impacts the design and development of the balance of plant (BOP) of the fuel cell, an image of which is shown in Figure 26:

**Figure 26:**
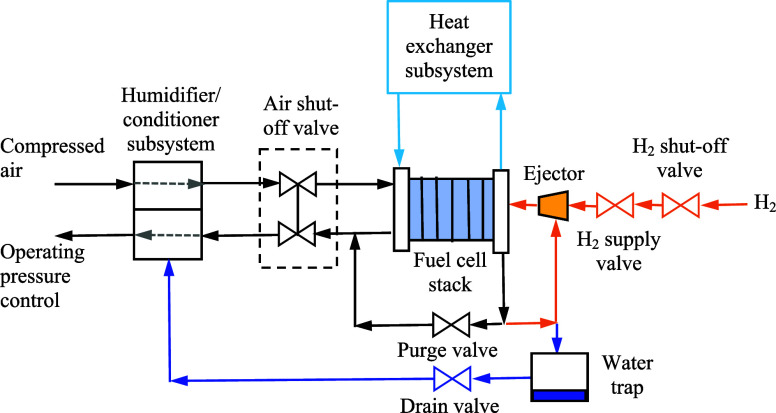
Sample fuel cell balance of plant.^27^

The two main modes of water transport across the fuel cell membrane are electro-osmotic drag and back diffusion. In typical fuel cell operation, the anode stoichiometry is greater than one to humidify unused hydrogen. The hydrogen is humidified through back diffusion, and back diffusion is dependent on the concentration gradient and hydration state of the membrane. To control the concentration gradient across the membrane, a humidifier is added to the cathode side, as shown in Figure 26 (to left of the fuel cell stack). If the electro-osmotic drag across the membrane is higher than predicted, it will lead to too much liquid water on the cathode side, possibly introducing premature flooding of the fuel cell, which would lower discharge voltage and increase heat generation. If the electro-osmotic drag is lower than predicted, then the membrane may dry out because there is not enough water on the cathode side to support back diffusion. The other components that are impacted by the total water flow are the injectors and ejectors on the anode side of the fuel cell (right side of the fuel cell stack in Figure 26). If the back diffusion is not sufficient, the recycled hydrogen will be dry, and this will cause the membrane to dry out, and the Ohmic losses of the fuel cell will thus increase. The total water flow across the membrane impacts both the cathode humidifier along with the anode injector and ejector. This is the main reason the back diffusion and electro-osmotic drag coefficients need to be accurately quantified to accurately model the water transport across the fuel cell and properly size all components.

The electro-osmotic drag coefficient and back diffusion coefficient will also impact the anode stoichiometry. On the anode side of the fuel cell, the hydrogen stoichiometry is always greater than one because the unused hydrogen is humidified via back diffusion and is mixed with the dry hydrogen via the injector/ejector assembly. If the actual back diffusion deviates from the modeled, then the anode stoichiometry will have to be adjusted to prevent too much water moving to the cathode. This is done by adjusting how much humidified hydrogen is sent to the ejector. The improved water flow models will impact the humidifier, injector, and ejector, and this will improve the fuel cell balance of plant design as well as the fuel cell design.

In the current research, it is shown that when the total water flow data trendline is not forced through the origin, for instance in the case of the Ye and Wang,^3^ the back diffusion component of the total water flow can be quantified and its direction established. In ref 3, the back diffusion was working against the electro-osmotic drag to some degree, and as a result the data trendline forced through the origin underreported electro-osmotic drag. To force the trendline of the total water flow data through the origin implicitly assumes other drivers are completely negligible. There are various fluid drivers from gradients of voltage, concentration, temperature, and pressure (Figure 9). Several of these may be present even to a small extent, and their influence could be masked by other drivers. Based on the prior data calculated with the current method, it was found that back diffusion opposed electro-osmotic drag, which resulted in a lower electro-osmotic drag coefficient compared to what was reported in the prior research. This opposing effect was also confirmed by Biesheuvel and Dykstra^23^ stating that electro-osmotic flow experiment necessitating multiple experiments.

The other fluid driver that was likely present in high relative humidity cases is osmotic distillation (a transport mechanism of liquid water across hydrophobic membranes due to phase change). The data analysis here was able to quantify the unique contribution of electro-osmotic drag from the total water flow data, but there was not enough information to estimate the osmotic distillation. The osmotic distillation portion of the total water flow could not be quantified because the osmotic distillation transfer coefficient is not known. An additional experiment is needed where liquid water is present on one side of the membrane and water vapor is present on the other. The vapor pressure on both sides would also be needed along with the temperature, as osmotic distillation will cause a thermal gradient due to the vaporization and condensation. This confirms what Biesheuvel and Dykstra^23^ stated—that an electro-osmotic flow experiment necessitates multiple experiments, including one without applied current. Although the method outlined here mines more information from available total water flow experimental data, many of these data sets lack enough detail to find tertiary drivers.

The current method outlined here showed that electro-osmotic drag coefficient in the Ye and Wang^3^ data did decrease with current density, where the prior research claimed it to be constant. The back diffusion component of the total water flow was not negligible in most of the prior data, and some of the data showed back diffusion playing a significant role. As the back diffusion was shown here to be working against electro-osmotic drag, prior research underreported the magnitude of the electro-osmotic drag. The current calculations would then be more detailed and accurate.

Park and Caton^28^ performed experiments with the objective to determine the electro-osmotic drag coefficient under variable current density, and they found that the electro-osmotic drag coefficient decreases with increasing current density. They proposed two theories: The first is that water formation on the cathode increases back diffusion. The second is that with increased current density, more protons are transported to the cathode, but with increased back diffusion, the net number of water molecules decreases.

With the proposed method outlined here, electro-osmotic drag and back diffusion experiments can be re-examined in more detail and with fewer assumptions, and the experimental results can now be compared even perhaps to the coefficient empirical models chosen in many prior works. With a better understanding of the electro-osmotic drag and back diffusion coefficient, the total water flow across the membrane can be more accurately modeled, potentially leading to better control strategies to optimize fuel cell operation.

## Nomenclature

*a*_o_: empirical coefficient from trendline fit (mol/s m^2^)
*a*_1_: empirical coefficient from trendline fit (mol/C)
*a*_2_: empirical coefficient from trendline fit (mol m^2^/(s A^2^))
*D*, *D*(λ): back diffusion coefficient (m^2^/s)
d*c*/d*x*: concentration gradient (mol/m^4^)
EW: equivalent weight of membrane (kg/mol)
*F*: Faraday’s constant 96485 C/mol
*I*: current (A)
*j*: current density (A/m^2^)
*J*: mass flux (mol/s m^2^)
*J*_q_: heat flux (W/m^2^)
*J*_BD_: back diffusion flow across the membrane (mol/s m^2^)
*J*_EOD_: electro-osmotic drag flow across the membrane (mol/s m^2^)
*J*_m_: mass flux across the membrane (mol/s m^2^)
*J*_TOTAL_: total water Flow across the membrane (mol/s m^2^)
*ṁ*_i_: mass flow of water inlet to fuel cell (mol/s)
*ṁ*_o_: mass flow of water outlet to fuel cell (mol/s)
*n*_d_: electro-osmotic drag coefficient (mol H_2_O/mol H^+^)
**Subscripts**
RBD: ration of back diffusion to total water flow
REOD: ratio of electro-osmotic drag to total water flow
**Greek symbols**
β: total water transfer coefficient (mol H_2_O/mol H^+^)
λ: hydration state of the membrane (unitless)
$\frac{d\lambda }{dx}$: concentration gradient represented by hydration state of the membrane difference (1/m)
Δ*E*: voltage difference (V)
Δ*P*: pressure difference (Pa)
Δ*T*: temperature difference (°C)
ρ: density of the dry membrane (kg/m^3^)
